# Fertility status perception, fertility preservation and desire to have children in cancer survivors: French VICAN survey

**DOI:** 10.4155/fsoa-2018-0018

**Published:** 2018-10-05

**Authors:** Margaux Jegaden, Anne-Déborah Bouhnik, Marie Préau, Marc-Karim Bendiane, Patrick Peretti-Watel, Julien Mancini, Blandine Courbiere

**Affiliations:** 1 Department of Obstetrics, Gynecology & Reproductive Medicine, Plateforme Cancer et Fertilité ONCOPACA-Corse/CECOS, APHM, La Conception Hospital, Marseille, France; 2 Aix Marseille Univ, INSERM, IRD, SESSTIM, Sciences Economiques & Sociales de la Santé &Traitement de l'Information Médicale, Marseille, France; 3 GRePS Lyon 2 Université, University of Lyon, Bron, France; 4 ORS PACA, Southeastern Health Regional Observatory, Marseille, France; 5 Aix Marseille Univ, APHM, INSERM, IRD, SESSTIM, Sciences Economiques & Sociales de la Santé & Traitement de l'Information Médicale, Hop Timone, BioSTIC, Biostatistique et Technologies de l'Information et de la Communication, Marseille, France; 6 Aix Marseille Univ, IMBE UMR7263, CNRS, IRD, Avignon Université, Marseille, France

**Keywords:** fertility counseling, fertility preservation, fertility status, French cancer survivors, gamete preservation, health politic, oncofertility, parenthood, VICAN, young adult cancer survivors

## Abstract

**Aim:**

To report fertility status perception, fertility preservation and desire to have children in French cancer survivors 2 and 5 years after diagnosis.

**Methods:**

A total of 427 women and 115 men self-reported treatment-induced infertility, fertility status, access to gamete conservation, desire to have children and pregnancy/live births.

**Results:**

A total of 96.5% of men and 92.9% of women were thought to be fertile at diagnosis and 38% desired to have children. A total of 57.8% of men and 67.4% of women declared that no fertility preservation had been discussed before treatment. After 2 years, 26.8% of patients still desired to have children. After 5 years, 18 live births have been reported.

**Conclusion:**

Despite a legal obligation and technical progress, there is a lack of information given to patients.

## Lay abstract

We conducted a study on the status of fertility counseling and preservation before cancer treatment in patients under 40 years old. Despite a legal requirement in France and advancements in the field, we demonstrate that there remains a lack of information given to patients and a low rate of fertility preservation.

In France, over 6% of cancers affect people between 15 and 44 years old [1]. With advances in therapies, the cancer survival rate has increased. Consequently, physicians and oncologists need to be aware of the side effects of oncologic treatment, such as gonadotoxicity [2,3]. Gonadal damage is dependent on the patient's fertility potential prior to oncologic treatment and on the type of treatment used. It is understood that the most gonadotoxic agents are radiotherapy (total body irradiation or pelvic radiation) and certain chemotherapeutic agents such as alkylating agents and platinum derivatives [4,5]. Patients are often treated by a combination of chemotherapy agents, making it difficult to accurately predict the consequences on one's fertility potential [6].

Fertility preservation (FP) can lead to better coping with diagnosis and less psychological distress during treatment [7]. Moreover, the possibility of having children is a determinant factor of the quality of life [7]. Oncofertility is a fairly recent concept, which includes both oncology and reproductive medicine, and aims to give a maximal chance of FP without any significant impact and delay in oncologic outcome. The FP was first formally inscribed into French Law in 2006 stating, *“Everybody for whom a medical treatment could impair fertility or for whom fertility could be prematurely altered, can have cryopreservation of their gametes or germinal tissue, with the aim to use them for Assisted Reproductives Technologies to help restore their own fertility”* [8]. Thus, in France, fertility risk counseling is mandatory, and the cost of FP techniques is entirely covered by the public French healthcare system. The American Society of Clinical Oncology has published comparable recommendations on oncofertility [9]. Futhermore, in 2007 the Cancer Public Health Policy from the French National Cancer Institute highlighted the oncofertility concept [10]. In 2013, (action 21.3), French Public Health Policy placed an emphasis on improving the access to fertility techniques for all young French people with cancer [11].

For men, sperm freezing has proven its effectiveness since the report of the first pregnancy resulting from intrauterine insemination of frozen sperm in 1953 [12]. Other alternatives to cryopreservation of ejaculated sperm include cryopreservation of surgically extracted spermatozoa in cases of azoospermia or cryopreservation of testicular tissue [6]. Female FP is limited by age and ovarian reserve at the time of diagnosis [13]. Cobo *et al*. showed that the age and the number of vitrified oocytes are the main prognostic factors to predict the chance of live birth after remission. Before 35 years, the probability of having a live birth with ten vitrified oocytes was estimated at 60.5%, but this rate decreased to 29.7% after 35 years with the same number of vitrified oocytes [14]. Oocyte cryopreservation by vitrification is for now the standard FP method after puberty [9,15]. However, this technique was authorized by French Law only in July 2011. Oocyte vitrification tends to replace *in vitro* fertilization with embryo conservation, owing to the similar live birth rates [16]. Ovarian transposition in case of pelvic irradiation can be offered [17]. Ovarian tissue cryopreservation and oocyte *in vitro* maturation are the only FP methods available before emergency chemotherapy, but they are still considered experimental techniques by the American Society for Reproductive Medicine and French law [6,18].

A previous national French survey was conducted in 2004–2005 by Mancini *et al*. with data from more than 10 years ago and before the updating of the French law in 2006, this study reported that 30% of women and 13% of men declared that they had not been informed about the risk of infertility before treatment [19]. When Mancini *et al*. asked them 2 years after cancer diagnosis about their fertility, 37% of women (<45 years old) and 30% of men (<71 years old) believed their cancer treatment induced infertility. Moreover, Mancini *et al*. reported that the localization and prognosis of cancer were not critical factors for the desire to have children, but did not directly ask patients about it [20]. The infertility risk of each cancer had been not evaluated, and to date, in France, there is no extensive study that questions patients on their changing desires and the influence of their cancer.

The objective of this study was to report on advancements in perception 5 years after the inscription of FP into French law. In addition, it sought to report on the access to a standardized FP program counseling procedure and the desire to have children in young adult French cancer survivors 2 and 5 years after their cancer diagnosis.

## Methods

### Data source

Implemented in 2012 and 2015, the national VICAN (‘vie après le cancer’) survey studied actual state life in cancer survivors 2 and 5 years after diagnosis. As described in a previous publication [21], the VICAN survey targeted men and women diagnosed between January and June 2010 and registered in the long-duration disease file of the National Health Insurance file of one of the three main French health insurance schemes, which cover >90% of the population. It was restricted to 12 cancer sites with good, intermediate or poor prognosis, accounting for 88% of cancer incidence in France. Eligibility was restricted to French-speaking patients diagnosed with their first malignancy and living in France for at least 2 years. The first data collection was conducted in 2012, 2 years after diagnosis, and included 4349 participants (global response rate 43.7%) [21]. The second data collection in 2015 involved a second interview of 2009 individuals who originally participated in the survey in 2012.

The study methodology was designed and performed in accordance with the Helsinki declaration, and was approved by the three French national ethics commissions: CCTIRS (Comité Consultatif sur le Traitement de l'Information en Matière de Recherche dans le Domaine de la Santé, study No. 11–143), ISP (Institute of Public Health, study No. C11-63) and CNIL (French Commission on Individual Data Protection and Public Liberties, study No. 911290).

### Study population

For the present study, only participants aged between 18 and 40 years old at time of diagnosis (the age limit for women to access fertility preservation) were included, which accounted for 585 participants interviewed in 2012 and 267 in 2015. Of those not interviewed in 2015, 36 were identified as having become deceased between questionnaires.

### Questionnaires

The patient questionnaire dealt with many topics including demographic background, socioeconomic status, living conditions, treatments received and perceived side effects. The first questionnaire 2 years after diagnosis also included items related to FP such as fertility status at diagnosis/2 years after, desire to have children before cancer and its influence on treatment choices, access to gamete conservation before treatment initiation and desire to have children at diagnosis and at the time of the survey. The second questionnaire, 5 years after diagnosis, also explored fertility; desire to have children and pregnancies/births since diagnosis.

### Medical characteristics

We combined three sources of data (patient questionnaires VICAN 2 and 5, medical surveys completed by physicians who initiated cancer treatment and national medico-administrative databases) to define the treatments received: surgery, chemotherapy regimen, radiotherapy and endocrine therapy [21].

### Statistical methods

χ^2^- and Student's *t*-tests were used for univariate comparisons. Then, to identify the factors independently associated with the suggestion of FP, a binary logistic regression model was used. A stepwise procedure was used to select statistically significant factors in a multivariate model (entry threshold; p < 0.20). Only variables remaining associated with the outcome with a p-value < 0.05 were finally kept in the model. All first-order interactions between associated variables were checked.

## Results

Participants were mostly women (79.5%), aged between 30 and 39 years old at diagnosis (70.3%). Most of them had breast cancer (46.9%). The most common localizations of cancer for both men and women were thyroid (15.6%), melanoma (12.1%) and non-Hodgkin lymphoma (NHL) (10.6%). A total of 56% of patients reported to have been treated by chemotherapy and 48% by radiotherapy, and 30.8% of women received a hormonal treatment (Table 1).

**Table 1.** **Sample characteristics: VICAN survey, n = 585.**

**Sample characteristics**	**Total number = 585 n (%)**	**Men number = 120 n (%)**	**Women number = 465 n (%)**	**p-value**
Age at diagnosis 18–29 30–39 40	85 (14.5) 411 (70.3) 89 (15.2)	22 (18.3) 86 (71.7) 12 (10.0)	63 (13.5) 325 (69.9) 77 (16.6)	0.120

Education higher than high school certificate No Yes	171 (29.2) 414 (70.8)	49 (40.8) 71 (59.2)	122 (26.2) 343 (73.8)	0.002

Children^†^ 0 1 2 or more	150 (25.6) 133 (22.7) 302 (51.6)	39 (32.5) 36 (30.0) 45 (37.5)	111 (23.9) 97 (20.9) 257 (55.3)	0.002

Localization of cancer Breast Lung Colorectal Upper Aerodigestive Tract Kidney Thyroid Melanoma NHL Cervix	218 (37.3) 11 (1.9) 51 (8.7) 19 (3.2) 26 (4.4) 91 (15.6) 71 (12.1) 62 (10.6) 36 (6.2)	– 4 (3.3) 21 (17.5) 11 (9.2) 14 (11.7) 14 (11.7) 33 (27.5) 23 (19.2) –	218 (46.9) 7 (1.5) 30 (6.5) 8 (1.7) 12 (2.6) 77 (16.6) 29 (6.2) 48 (10.3) 36 (7.7)	<0.001

Underwent chemotherapy No Yes	255 (43.6) 330 (56.4)	57 (47.5) 63 (52.5)	198 (42.6) 267 (57.4)	0.333

Underwent radiotherapy No Yes	304 (52.0) 281 (48.0)	89 (74.2) 31 (25.8)	215 (46.2) 250 (53.8)	<0.001

Was prescribed hormonal treatment (women only) No Yes			322 (69.2) 143 (30.8)	

Cancer progression in 2 years after diagnosis No Yes	481 (82.2) 104 (17.8)	85 (70.8) 35 (29.2)	396 (85.2) 69 (14.8)	<0.001

Was fertile at diagnosis (men only) No Yes Missing		3 (2.5) 116 (96.7) 1 (0.8)		

Was fertile at diagnosis (women only) No, sterile No, menopausal No, pregnant Yes missing			7 (1.5) 4 (0.9) 18 (3.9) 432 (92.9) 4 (0.8)	

Among fertile participants	n = 548	n = 116	n = 432	

Reported a desire to have children at diagnosis No Yes, probably Yes, certainly Missing	339 (61.9) 64 (11.7) 144 (26.3) 1 (0.2)	69 (59.5) 8 (6.9) 38 (32.8) 1 (0.9)	270 (62.5) 56 (13.0) 106 (24.5) –	0.309

The desire to have children influenced treatment^††^ Yes No Did not know	27 (13.0) 177 (85.1) 4 (1.9)	3 (6.5) 40 (87.0) 3 (6.5)	24 (14.8) 137 (84.6) 1 (0.6)	0.269

Did a cryoconservation at diagnosis Yes No, not proposed No, not wanted missing	36 (6.6) 358 (65.3) 148 (27.0) 6 (1.1)	24 (20.7) 67 (57.8) 24 (20.7) 1 (0.9)	12 (2.8) 291 (67.4) 124 (28.7) 5 (1.2)	<0.001

Reported to be sterile 2 years after diagnosis Does not know Yes No Missing	79 (14.4) 76 (13.9) 391 (71.4) 2 (0.4)	28 (24.1) 9 (7.8) 78 (67.2) 1 (0.9)	51 (11.8) 67 (15.5) 313 (72.5) 1 (0.2)	0.001

Reported a desire to have children 2 years after diagnosis No Yes, in the coming months Yes, in a longer term Missing	391 (71.3) 45 (8.2) 102 (18.6) 10 (1.8)	68 (58.6) 16 (13.8) 27 (23.3) 5 (4.3)	323 (74.8) 29 (6.7) 75 (17.4) 5 (1.1)	0.0149

Cancer influenced the desire to have children Yes, by cancelling or reporting it Yes, by reinforcing it No Missing	168 (30.7) 31 (5.7) 342 (62.4) 7 (1.3)	21 (18.1) 8 (6.9) 83 (71.6) 4 (3.5)	147 (34.0) 23 (5.3) 259 (60.0) 3 (0.6)	0.002

^†^Total 2 years after diagnosis.

^††^Among participants who reported a desire for a child at diagnosis (n = 208).

NHL: Non-Hodgkin lymphoma.

At the time of cancer diagnosis, 96.7% (n = 116) of men in the study thought they were fertile. Among them, 39.7% reported to have had a desire for a child before cancer. For 32.8% of them, their desire was strong. Among women, 18 (3.9%) were pregnant, 1.5% reported being sterile and 0.9% reported to be menopausal. As with men, only women who thought themselves fertile (92.9%; n = 432) were asked about their desire to have children at diagnosis. Among them, 37.5% declared such desire, among which 24.5% had a strong desire. The rate of desire for a child increased up to 71.2% among individuals aged under 35 years and in couples with no children. Moreover, 6.5% of men and 14.8% of women declared that desire for children had influenced the treatment. Indeed, women with a desire for children received surgery (87.5 vs 93.4%; p = 0.307), chemotherapy (70.8 vs 56.2%; p = 0.180) and/or irradiation (75.0 vs 56.2%; p = 0.084) as often as women without such desire.

Concerning FP counseling, 57.8% of men and 67.4% of women reported that no FP counseling had been provided to them before cancer treatment (Table 2).

**Table 2.** **Factors associated with fertility preservation, univariate analyses: VICAN survey, n = 542.**

**Factors associated with fertility preservation**	**Was proposed cryoperservation**			**p-value**

	**Total number = 542 n (%)**	**No number = 358 n (%)**	**Yes number = 184 n (%)**	
Gender Male Female	115 (21.2) 427 (78.8)	67 (58.3) 291 (68.1)	48 (41.7) 136 (31.9)	0.047

Age at diagnosis (mean [SD])	35.0 (4.9)	34.6 (5.0)	35.6 (4.6)	0.026

Education higher than high school certificate No Yes	150 (27.7) 392 (72.3)	96 (64.0) 262 (66.8)	54 (36.0) 130 (33.2)	0.533

Children^†^ 0 1 2 or more	138 (25.5) 126 (23.2) 278 (51.3)	83 (60.1) 97 (77.0) 178 (64.0)	55 (39.9) 29 (23.0) 100 (36.0)	0.009

Reported a desire to have children at diagnosis No Yes	336 (62.0) 206 (38.0)	200 (59.5) 158 (76.7)	136 (40.5) 48 (23.3)	<0.001

Lived as a couple at diagnosis No Yes	474 (87.5) 68 (12.5)	310 (65.4) 48 (70.6)	164 (34.6) 20 (29.4)	0.398

Underwent chemotherapy No Yes	235 (43.4) 307 (56.6)	180 (76.6) 178 (58.0)	55 (23.4) 129 (42.0)	<0.001

Localization of cancer Breast Lung Colorectal UADT Kidney Thyroid NHL Melanoma Cervix	203 (37.5) 7 (1.3) 49 (9.0) 14 (2.6) 25 (4.6) 86 (15.9) 61 (11.3) 65 (12.0) 32 (5.9)	131 (64.5) 5 (71.4) 35 (71.4) 9 (64.3) 18 (72.0) 65 (75.6) 21 (34.4) 52 (80.0) 22 (68.8)	72 (35.5) 2 (28.6) 14 (28.6) 5 (35.7) 7 (28.0) 21 (24.4) 40 (65.6) 13 (20.0) 10 (31.3)	<0.001

Underwent radiotherapy No Yes	279 (51.5) 263 (48.5)	187 (67.0) 171 (65.0)	92 (33.0) 92 (35.0)	0.622

Was prescribed hormonal treatment (women only) No Yes	410 (75.6) 132 (24.4)	271 (66.1) 87 (65.9)	139 (33.9) 45 (34.1)	0.968

Fertility preserved^†^ No Yes	204 (37.6) 338 (62.4)	131 (64.2) 227 (67.2)	73 (35.8) 111 (32.8)	0.483

^†^Total 2 years after diagnosis.

NHL: Non-Hodgkin lymphoma; SD: Standard deviation.

Concerning FP, 20.7% of men reported having performed sperm banking before cancer treatment. The majority of them had a NHL (75.0%), 8.3% had a thyroid cancer, 12.5% a colorectal cancer and 4.2% an upper GI tract cancer. Of the 79.3% of men who did not perform cryoconservation, 20.7% reported having refused it. Among women, only 2.8% of them reported to have access to ovarian tissue cryopreservation, oocyte cryopreservation or emergency *in vitro* fertilization for embryo freezing prior to treatment. Most of these women were diagnosed with breast cancer (58.3%) and NHL (25.0%), while 8.3% had cervical cancer or thyroid cancer. Of the 77.2% of women who did not perform cryoconcervation, 28.7% reported having refused it.

In univariate analysis, men were significantly more often proposed to have FP counseling than women (41.7 vs 31.9%; p = 0.04), as well as older patients, and those with no children (Table 2). Paradoxically, reporting a desire to have children was associated with fewer suggestions of FP from oncologists (23.3 vs 40.5%; p < 0.001). However, men with such desire were more likely to undergo a FP technique (11.2 vs 3.9% among participants with no desire to have children at diagnosis; p < 0.001). Others factors significantly associated with the suggestion of FP counseling after multiple adjustment were the absence of children, NHL diagnosis and chemotherapy as treatment (Table 3).

**Table 3.** **Factors associated with fertility preservation, multivariate analysis: VICAN survey, n = 542.**

Factors associated with fertility preservation	**Adjusted OR**	**CI 95%**	**p-value**
Children^†^ 0 1 2 or more	1 0.4 0.6	(0.2–0.7) (0.4–1.0)	0.008 0.002

Reported a desire to have children at diagnosis No Yes	1 0.4	(0.2–0.6)	<0.001

Underwent chemotherapy No Yes	1 2.0	(1.3–3.0)	<0.001

Localization of cancer: NHL Others	3.8 1	(2.1–7.0)	<0.001

^†^Two years after diagnosis.

CI: Confidence interval; NHL: Non-Hodgkin lymphoma; OR: Odds ratio.

A total of 2 years after diagnosis, of the 116 men who thought they were fertile at diagnosis, 67.2% believed they remained fertile after treatment, 7.8% thought they were sterile, and the rest were uncertain about their fertility status. Moreover, 37.1% of men reported a desire to have children. Among the 432 women who thought they were fertile at diagnosis, 67 (15.5%) thought themselves to be menopausal 2 years after treatment and 51 (11.8%) did not know about their fertility status. A total of 2 years after diagnosis, 72.5% of women still believed themselves to be fertile, and 24.1% reported a desire to have children. The desire to have children was stronger among men (Table 1), and women more often reported that cancer had a negative influence on their desire (34.0 vs 18.1% among men, p = 0.002).

**Figure 1.**
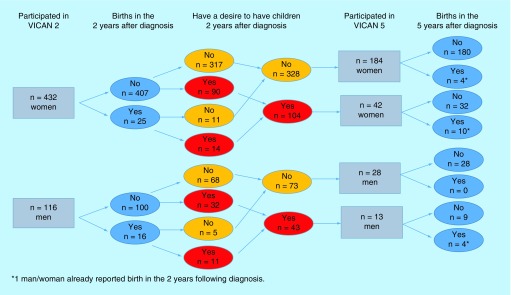
**Flow chart representing birth rates since cancer diagnosis: VICAN survey.** VICAN survey: Vie après le cancer survey.

A total of 5 years after diagnosis, 267 participants answered the 5-year questionnaire. After having excluded the 36 participants who were deceased between 2012 and 2015, we compared them with the 282 nonrespondents: respondents were more often women and had a high level of education (Figure 1). No other difference was found between respondents and nonrespondents. The majority of the 267 respondents (n = 226, 84.6%) were women and only 41 were men. A total of 13 of the 41 men reported a desire to have children 2 years after diagnosis. A total of 3 years later, of these 13 men, three reported infertility. Five men still reported a desire for a child, and the remaining men no longer had a desire to have children. Among the 226 women, 42 reported a desire to have children 2 years after diagnosis. A total of 5 years after diagnosis, of these 42 women, three reported having no information regarding their fertility, eight reported infertility, five reported that cancer affected their desire to have children. Ten women still reported a desire to have children 5 years after diagnosis, and the remaining six women no longer had that desire.

Concerning birth rates since the diagnosis, on the 432 women who declared themselves fertiles at the time of diagnosis, 25 reported having a child in 2 years following their diagnosis, with 36% of them during anticancer treatment. Of the 432 women, 104 declared having a desire for a child 2 years after diagnosis. A total of 42 of them answered the 5-year questionnaires; ten reported having a child since diagnosis, and three had a consultation with a specialist in reproductive medicine at the end of their treatments. Of the 116 fertile men at the time of diagnosis, 16 had a child in 2 years following, of which 15 were conceived naturally. A total of 43 of them declared a desire for a child 2 years after diagnosis and 13 of them answered 5 years later, of which four reported having a child since diagnosis, including only one man who had a consultation with a reproductive medicine specialist (Figure 1).

## Discussion

Total 5 years after the initial study of Mancini *et al*. and 8 years after the French law on FP counseling, our study observed that there is still a lack of FP counseling and access to FP techniques before cancer treatment [19]. Despite a national French Public Health Policy, only 20.7% of men under age 40 reported to have performed sperm freezing before cancer treatment and FP was used by only 2.8% of women. In our cohort, 57.8% of men and 67.4% of women declared that no FP techniques had been discussed before cancer treatment. Despite the fact that the first auto questionnaire was administered only 2 years after cancer diagnosis, the used methodology could induce recall bias. In the context of the overwhelming psychological distress of a new cancer diagnosis, the FP counseling may have been provided but not internalized by the patient and therefore not reported. Hence, written information regarding FP should also be provided to allow patients time to digest all information. Today, to provide better access to FP counseling to all French cancer patients is one of the objectives of the French National Anti-Cancer Strategy 2014–2019.

Despite being insufficiently offered, FP counseling was more often proposed to men than women (41.7 vs 31.9%; p = 0.047). Letourneau *et al*. reported that between 1993 and 2007 the rate of FP in women was 4% [22]. Indeed, semen is technically much easier to obtain than mature oocytes or ovarian tissue. Sperm banking is a routine technique, and FP techniques in women such as oocyte cryopreservation have only recently been optimized [9]. In France, oocyte vitrification is allowed by law only since July 2011. Patients surveyed here were diagnosed in 2010, and our study could not show the positive impact of the oocyte vitrification on the implementation of FP in women. Since 2012, a twofold increase of FP in women was observed in a French region [23]. Widespread access to FP would be interesting to ascertain in five more years as this may give adequate time to reflect a change in practice patterns. Moreover, gender effect was no longer significant after multiple adjustments because the men surveyed more often had an NHL and fewer children. Reasonably, another factor associated with proposition of FP counseling should be the desire to have children or not. At the time of diagnosis, 38% of patients reported to have a desire for a child. This rate seems very low, but logical if we consider that more than 70% of patients already had children at the time of diagnosis. Having or not having children already should probably be the most important factor for considering and discussing FP. In fact, it seems that this desire was not taken into account. Paradoxically, FP was advised more frequently to those who did not desire future childbearing at the time of diagnosis (40.5 vs 23.3%). Among men and women with FP discussions at time of diagnosis, 27% reported having refused the cryoconservation; this probably has to do with having children already. Furthermore, oncologists declared taking into account their patients’ desire to have children when they determine specific treatment options [24]. For the patient, this desire for a child has not influenced therapeutics choices for men (6.5%). However, 14.8% of women declared having asked their oncologist to change their treatment for one with lower gonadotoxicity. In practice, most of the time, treatments were more often adjusted than rejected. Other studies should be performed to verify that those modifications do not influence healing prognosis.

Moreover, our study has highlighted a lack of information given to patients about the gonadotoxicity of their treatment. It is noteworthy that 13.8% of men conceived spontaneously in 2 years after cancer despite the risk of DNA damage on spermatozoa described until 2 years after anticancerous treatment [25]. For women, 2.1% of them were pregnant during anticancer treatment, emphasizing the importance to prescribe and explain the necessity of an efficient contraception. A total of 2 years after cancer, 67.2% of men and 72.5% of women thought themselves to be able to procreate after their cancer treatment. These data are similar to the results previously observed in 2004 by Mancini *et al*. [19,20]. Fertility perception seems to be altered 2 years after diagnosis. Only 7.8% of men declared themselves sterile but the important rate of no answer on this question leads us to suppose that it is difficult for them to approach this topic, or they are unable to answer/unaware of the answer to this question about their fertility. Women were less uncertain about their fertility with more answering than men, and thought themselves more affected by infertility; only 60.6% thought themselves fertile after treatment. It would have been interesting to determine what factors lead women to their perception of their fertility status: restoration of menses or hormonal blood test. Presently, as recommended, we advise women to consult 1 year after the end of treatment to study postcancer ovarian function. We must provide patients with the maximum information on the possible consequences of treatments on gonadal function and a long term follow-up to manage infertility and/or endocrine dysfunction [26].

A total of 2 years after cancer, 37.1% of men and 24.1% of women still had the desire to have children and the frequency of desire was higher for patients under 35 years (around three quarters of them). This shows why it is important to identify the current barriers that could be responsible for the lack of implementation of FP. Possible barriers include fear of losing time and making the prognosis worse, altered patient health conditions, lack of time during consultation, refusal by patient, lack of interest at the time of diagnosis or psychological distress in a life-threatening situation. The main barrier is sometimes the oncologist, who may not discuss FP with the patient, which results in lost opportunities to discuss the future fertility concerns [27]. A previous French regional study conducted in 2012 reported that 54% of oncologists had never referred a patient for a FP specialized consultation before cancer treatment [23]. These data are concordant with the low FP activity of a French regional referential center with only 71 patients referred in 2 years [28]. Indeed, 58% of a cohort of French oncologists declared themselves to be not sufficiently trained in oncofertility [23]. In the present study, the subgroup of oncologists specialized in hematologic disease seemed to be better informed and more sensitive to FP, as 65.6% of patients with NHL had received fertility counseling. Data show an important disparity between cancer types as well: 24.4 and 35.5% of patients received fertility counseling for thyroid and breast cancer, respectively. A British study on oncologists’ practices was in line with these French data [29]. Obviously, oncologists need to be regularly informed. Solutions have been created to develop a FP network such as creating websites on oncofertility or accessibility to an oncofertility degree. Moreover, oncofertility needs to offer a standardized interdisciplinary specialized FP counseling procedure to help narrow the gap between reproductive medicine and oncology [30]. In 2012, 43% of oncologists declared that they had some technical difficulties to contact fertility specialists consequently, a French Regional Networks ‘Cancer & Fertility’ have been developed to help communication between oncologists and reproductive physicians [23].

After cancer diagnosis, a relatively large proportion of men (13.8%) and women (5.8%) have had a child, or had planned to have a child (37.1 and 24.1%, respectively). If we consider the rate of patients who thought themselves still fertile, we found no link between cancer characteristics and desire to have children. Consistent with our previous survey, a more severe prognosis does not seem to be an obstacle to the desire for a child [20]. Nonetheless, women (34.0%) and men (18.1%) declared a negative impact of cancer on their desires. One of the reasons for delaying a pregnancy project could be the desire of patients to be sure that their treatment will have no negative effect. On the other hand, 5.7% have declared a positive effect of cancer on their parenthood desire. Procreation after cancer could give a patient a means to return to a ‘normal life’ [31].

However, data from 5 years after diagnosis shows that the majority of desires to bear children at 2 years after diagnosis had not been granted. There were only ten births out of 42 women with a desire to have children, and four out of 13 for men.

There is a lot of drop out between the two questionnaires, which we can partially explain with patient's death, cancer relapse or address change. We can also suppose a disinterest or a determination from patients to forget about their disease 5 years after diagnosis. Even with many patients being lost to follow-up and the limits of the study design, this low rate of pregnancy was unexpected. Excluding the advanced age of patient and infertility, we can suppose that the desire to have a child was not so strong 2 years after diagnosis; this could be more reaction and attitude to cancer rather than real fertility determination. We can also explain this low rate by changing life goals, marital status and other factors. Moreover, the fact that only one man and three women have had a consultation with a specialist in reproductive medicine before having a child highlights once more the lack of follow-up and advisement of FP for patients with a new diagnosis of malignancy.

Before concluding, we must acknowledge several limitations. The VICAN survey shares the general limitations of any approach using self-administered questionnaires, which may be affected by recall bias as previously mentioned and social desirability bias. Moreover, our sample included a limited number of men compared with women, especially in the 5-year follow-up (n = 41). The large proportion of women in the VICAN 2 survey could be explained by the high incidence of breast cancer, especially in this age group, and its high survival rate (86% at 5 years) [1]. The high attrition in the 5-year follow-up was also due to the lower participation of men in the 5-year follow-up [32].

## Conclusion

Fertility potential is a major factor in determining the quality of life among young adult cancer survivors. At the time of the diagnosis, most patients under age 40 believe that they are able to procreate and have a family project. Despite a legal obligation to inform patients in France about the possible adverse gonadotoxic consequences on fertility with treatment, our results highlight a lack of information given to patients. Despite the physicians’ awareness, too few patients, especially women, were offered access to counseling and FP techniques. Some inequalities persist between men and women in spite of recent progress in FP for women with oocyte vitrification. Contrary to sperm banking, female FP will always be more complicated due to ovarian physiology and the age-related decline in fertility. Regardless of gender differences, information should be provided for all patients even when oocytes or ovarian tissue could not be cryopreserved. Oncologists should refer every patient to a reproductive medicine center before a gonadotoxic treatment. The best way to not forget this would be to develop in every cancer center a standardized multidisciplinary program that entails counseling on FP.

## Future perspective

The results of our study did not show real progress on patient fertility counseling and FP before cancer. But with the recent creation of a regional and national specialist network, access to information and fertility techniques have been increased. With the recent authorization of oocyte vitrification in France, and the encouraging results from ovarian cortex grafts, FP for women should be performed more often. It would be interesting to carry out a similar study in 5–10 years to show the impact of new medical advance.

**Summary points**

**Objective**

- To report fertility status perception, fertility preservation and desire to have children in French cancer survivors 2 and 5 years after diagnosis, respectively.

**Methods**

- A total 427 of women and 115 of men self-reported treatment-induced infertility, fertility status, access to gamete conservation, desire to have children and pregnancy/live birth.

**Results**

- A total of 96.5% of men and 92.9% of women thought themselves to be fertile at diagnosis and 38% desired to have children. A total of 57.8% of men and 67.4% of women declared that no fertility preservation had been discussed before treatment. After two years, 26.8% of men and women still desired to have children. After 5 years, 18 live births have been reported.

**Conclusion**

- Despite a legal obligation in France and technical progress for crypreserving gametes, there remains a perceived lack of information given to patients.
